# Detection of aspartyl aminopeptidase in atherosclerosis mice and clinical sample using an optical probe

**DOI:** 10.1016/j.mtbio.2025.102391

**Published:** 2025-10-08

**Authors:** Chenhui Zhou, Fangkun Yang, Chunyan Li, Hengyi Mao, Kai Wang, Jinhui Shang, Xiang Gao, Wenming He

**Affiliations:** a Department of Neurosurgery, The First Affiliated Hospital of Ningbo University, School of Medicine, Ningbo University, Ningbo, China; b State Key Laboratory of Pharmaceutical Biotechnology, School of Life Sciences, Nanjing University, Nanjing, 210023, China; c Department of Cardiology, The First Affiliated Hospital of Ningbo University, School of Medicine, Ningbo University, Ningbo, China; d Key Laboratory of Precision Prevention and Treatment for Atherosclerotic Diseases of Zhejiang Province, Ningbo, China

**Keywords:** *In vivo* imaging, Aspartyl aminopeptidase, Biomarker, Atherosclerosis diagnosis

## Abstract

Atherosclerosis (AS) remains a major global health concern, contributing significantly to cardiovascular morbidity and mortality. Recent research into enzymes like aspartyl aminopeptidase (DNPEP) has shed light on its potential role in regulating inflammation and protein metabolism, offering new avenues for targeted diagnostics and therapies. In light of these challenges, this study aims to develop a novel optical imaging probe (HD-DNPEP) specifically designed to detect DNPEP expression in atherosclerotic lesions. By addressing the current limitations in sensitivity, specificity, and in vivo detection, we seek to create a highly selective and sensitive imaging tool that can provide real-time, high-resolution insights into the role of DNPEP in atherosclerosis. This innovative approach has the potential in the diagnosis of atherosclerosis, enabling more precise intervention strategies and improving patient outcomes. In AS patients, the significantly higher DNPEP levels in the cerebral vasculature and CSF of AS patients suggest that DNPEP may serve as an important biomarker for detecting cerebrovascular arteriosclerosis.

## Highlights

• **Novel optical probe (HD-DNPEP)** developed for sensitive, specific detection of aspartyl aminopeptidase.
• **"Turn-on" fluorescence and photoacoustic (PA) signals**.
• **High selectivity and pH stability**: HD-DNPEP responds selectively to DNPEP in physiological conditions.
• **Cellular and in vivo validation**: Detects elevated DNPEP in foam cells (correlating with lipid accumulation).
• **Clinical relevance**: Confirmed significantly higher DNPEP levels in cerebrospinal fluid (CSF).

## Graphical abstract

The HD-DNPEP optical probe enables sensitive, real-time detection of DNPEP—a potential atherosclerosis biomarker—via turn-on fluorescence/PA signals, validated in cells, AS mice, and human clinical samples with high selectivity and biosafety, offering translational diagnostic potential.

**Figure:**
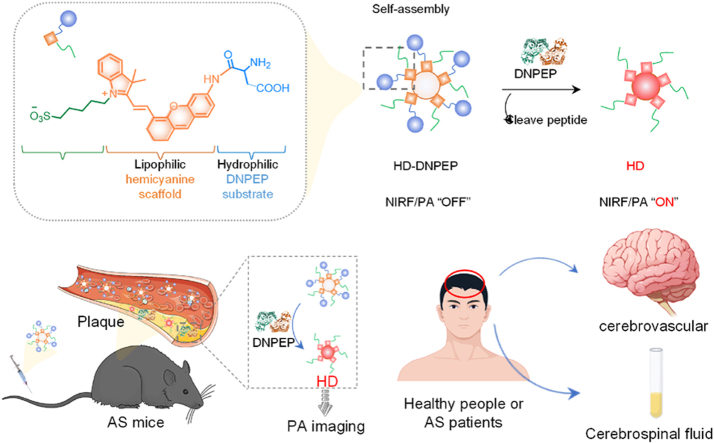

## 1 Introduction

Atherosclerosis is a chronic cardiovascular condition characterized by the accumulation of lipids, inflammatory cells, and fibrous elements within the arterial walls, leading to plaque formation and arterial narrowing [[1], [2], [3], [4]]. This process increases the risk of serious cardiovascular events, such as heart attack, stroke, and peripheral artery disease, making it a significant public health concern globally [[5], [6], [7]]. The progression of atherosclerosis is associated with endothelial dysfunction, oxidative stress, and the formation of advanced atherosclerotic lesions that impair blood flow [8,9]. Despite advancements in prevention and treatment, atherosclerosis remains one of the leading causes of morbidity and mortality worldwide, necessitating more effective diagnostic tools and therapeutic strategies to combat its impact [[10], [11], [12], [13], [14]].

Recent studies have highlighted the importance of various enzymes and biomarkers in the development and progression of atherosclerosis [10,[15], [16], [17]]. One such enzyme, aspartyl aminopeptidase (DNPEP), has gained attention for its involvement in the regulation of protein metabolism, cell signaling, and immune responses [18]. DNPEP has been shown to contribute to the inflammatory processes that play a central role in the pathogenesis of atherosclerosis [19]. Elevated expression of DNPEP in atherosclerotic lesions suggests its potential as a biomarker for the condition. By regulating the cleavage of aspartic acid-containing peptides, DNPEP may influence the stability of plaques and the progression of arterial damage. Understanding the specific role of DNPEP in atherosclerosis could pave the way for novel therapeutic targets and improved disease management [20,21].

In this study, we developed the HD-DNPEP probe by condensing HD-SO_3_H with DNPEP substrates, characterized by NMR and MS. The probe demonstrated excellent performance in detecting DNPEP, showing a "turn-on" fluorescent and photoacoustic (PA) signal with strong linear correlations (R^2^ = 0.992 for fluorescence, R^2^ = 0.993 for PA). It was highly sensitive, specific, and responsive within the physiological pH range, making it ideal for quantitative enzyme detection. In cellular experiments, it detected DNPEP in foam cells, with fluorescence intensity increasing with foam cell maturation. In vivo, the probe differentiated atherosclerotic plaques from healthy vessels using PA imaging and showed no significant toxicity. It also detected elevated DNPEP levels in serum, suggesting its potential for atherosclerosis detection and monitoring. Furthermore, we conducted clinical validation to complement our previous animal experiments, and the results were consistent with our prior findings. Specifically, we observed that the levels of DNPEP in the cerebral vessels and cerebrospinal fluid (CSF) of patients with arteriosclerotic disease were significantly higher than those found in the control group. This confirms that the elevated enzyme activity detected in animal models is also present in human patients, supporting the clinical relevance of our findings.

## 2 Materials

All chemical reagents were procured from Aladdin (Shanghai, China) and Sigma-Aldrich (St. Louis, MO, USA). Indole (Cat No. 1018212, Leyan, Shanghai, China). The hematoxylin and eosin (H&E) stain solution was purchased from Solarbio (Beijing, China). Fetal Bovine Serum (FBS) was obtained from NEST Biotechnology Co., Ltd. (China). Minimum Essential Medium (MEM) was purchased from absin (Shanghai) Biotechnology Co., Ltd. (abs9467-500 mL). Some cartoon components were from www.figdraw.com for model drawing.

## 3 Results

### 3.1 Design, synthesis, and response of HD-DNPEP

**Fig. 1:**
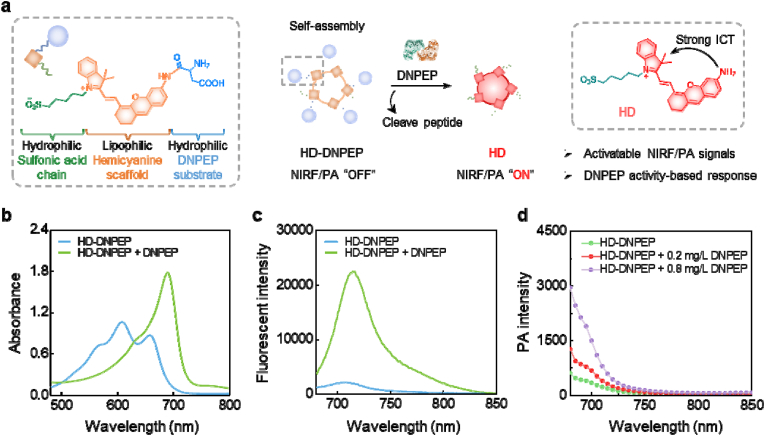
(a) Chemical structure and the responsive progress of HD-DNPEP to DNPEP enzyme. (b) Absorption spectra of HD-DNPEP before and after incubation with DNPEP. (c) Fluorescence spectra of HD-DNPEP before and after incubation with DNPEP. (d) PA spectra of HD-DNPEP before and after incubation with different concentrations of DNPEP.

HD-DNPEP probe was constructed by condensation of the HD-SO_3_H and the specific substrates of DNPEP, which consisted of the hemicyanine scaffold, a hydrophilic sulfonic acid chain, and a hydrophilic peptide [22]. The successful synthesizes of the PA probe were characterized by nuclear magnetic resonance (NMR) spectroscopy and mass spectrometry (MS) (Figures S1–S4). The amino of HD-DNPEP was caged with the peptide, which could inhibit the intramolecular charge transfer (ICT) effect, thus HD-DNPEP probe exhibited weak fluorescence and PA signal. In the unactivated state of HD-DNEPE, the amino group is modified by a peptide. The electron-withdrawing effect of the carbonyl group in the amide bond will lead to a significant reduction in the electron-donating effect of the amino group, thereby reducing the intramolecular charge transfer effect of HD-DNPEP. Notably, the peptide of HD-DNPEP probe could be specifically recognized by DNPEP, the amino group is released, thereby restoring the strong electron-donating ability and restoring the intramolecular charge transfer effect. The absorption spectrum redshifts, and the absorption increases at 700 nm, which leads to the absorption of more 700 nm light and the release of a sound signal, thus presenting strong PA and fluorescence signals, causing a “turn-on” fluorescent and PA signal (Fig. 1a).

Due to the simultaneous hydrophilic and hydrophobic fragments in HD-DNPEP, nanoparticles can be self-assembled in aqueous solution. Through dynamic light scattering, we found that the particle size of HD-DNPEP changed little before and after incubation with DNPEP, and was about 100 nm (Figure S5). In order to assess the performance of the probe, we carried out a comprehensive analysis of its absorption spectrum after introducing DNPEP, as shown in Fig. 1b. A noticeable decrease in absorption between 550 and 600 nm, along with the appearance of a distinct peak near 700 nm, was observed. This change implies the occurrence of enhanced intramolecular charge transfer (ICT). The observed spectral shift suggests significant alterations in the probe's molecular structure, either in its conformation or electronic state, likely facilitating the ICT process. The emergence of the peak at 700 nm indicates a change in the probe's electronic configuration, which is probably a result of its interaction with DNPEP.

Fluorescence spectroscopy confirmed HD-DNPEP's interaction with DNPEP, showing a sharp intensity increase upon enzyme addition (Fig. 1c). This likely stems from energy state changes upon binding, boosting emission. Similarly, photoacoustic (PA) signals surged at 680–720 nm with DNPEP (Fig. 1d), correlating with enzyme concentration, enabling quantitative detection. Both fluorescence and PA data consistently validated the probe's precision. The results demonstrate HD-DNPEP's reliability for DNPEP detection, with electronic/conformational changes enhancing selectivity—making it a promising tool for enzyme analysis.

### 3.2 Response of HD-DNPEP to DNPEP

**Fig. 2:**
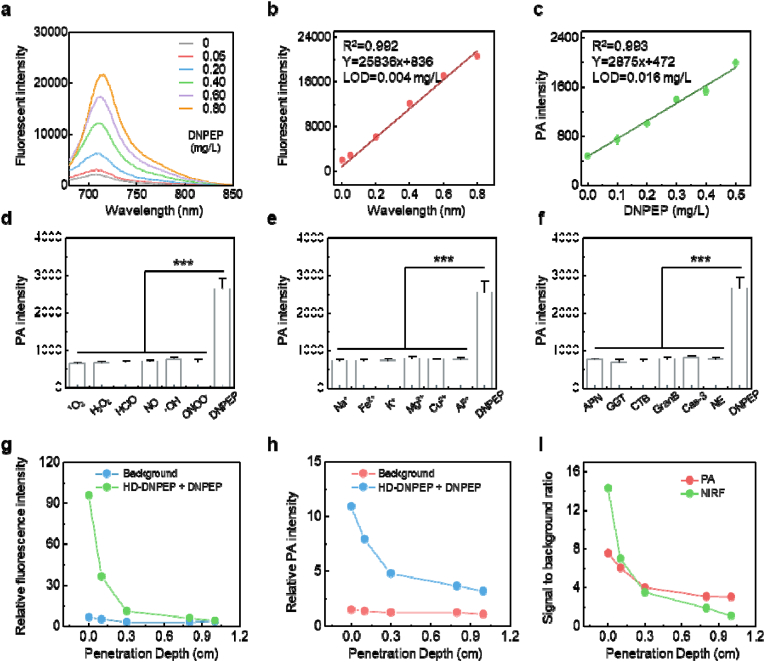
(a) Fluorescence spectra of HD-DNPEP after incubation with various concentrations of DNPEP (0 to 0.8 mg/L). (b) The linear relationship of fluorescence of HD-DNPEP and DNPEP activity in (a). (c) The linear relationship of PA intensity of HD-DNPEP and DNPEP activity. (d, e, and f) PA intensity of HD-DNPEP after incubation with various species. APN: Aminopeptidase N; GGT: Gamma-Glutamyl Transferase; CTB: Cathepsin B; GranB: Granzyme B; Cas-3: Caspase 3; NE: Neutrophil elastase. (g and h) Fluorescence (g) and PA (h) signals of HD-DNPEP after incubation with DNPEP covered with different thicknesses of the chicken breast. (i) The signal background ratio (SBR) of fluorescence and PA imaging in (g and h). Results were expressed as the mean ± s.d. Statistical significance (ns: P > 0.05, ∗P < 0.05, ∗∗P < 0.01, ∗∗∗P < 0.001).

We next investigated the responsiveness of the HD-DNPEP probe to the DNPEP enzyme. Upon exposure to DNPEP, a marked enhancement in the fluorescence emission of the HD-DNPEP probe was observed. Importantly, this fluorescence increase was accompanied by the emergence of a distinct peak at 710 nm, signaling the probe's activation (Fig. 2a). This fluorescence shift is crucial for unraveling the interaction mechanisms between the HD-DNPEP probe and DNPEP, providing valuable insights into the probe's potential as a highly sensitive sensor for enzyme activity.

Moreover, we examined the relationship between the fluorescence intensity at 710 nm and DNPEP enzyme activity. A strong, positive linear correlation was evident, as depicted in Fig. 2b, with an impressive coefficient of determination (R^2^ = 0.992). This finding suggests that the fluorescence response of the HD-DNPEP probe is sensitive to changes in DNPEP activity, positioning it as a dependable measure for enzyme activity levels within the tested concentration range. At the same time, we used a In Vivo Imaging Systems (IVIS) instrument to test the fluorescence response ability of HD-DNPEP (Figure S6). We found that with the increase of DNPEP concentration, the fluorescence signal of the probe gradually increased, which was consistent with the results of fluorescence spectrum test.

In addition, we explored the photoacoustic (PA) response of the HD-DNPEP probe to further assess its potential as a DNPEP activity sensor. The HD-DNPEP probe showed a strong linear PA signal increase with DNPEP concentration (0–0.5 mg/L, R^2^ = 0.993, Fig. 2c) with a LOD of 0.016 mg/L, confirming its utility for real-time, quantitative enzyme monitoring.

We systematically assessed the performance of HD-DNPEP across a variety of physiological settings, with a specific emphasis on how pH variations influence its responsiveness. The probe performed optimally at physiological pH (7.0–7.4, Figure S7) and exhibited high specificity for DNPEP, unaffected by metal ions or other enzymes (Fig. 2d–f, S8-S10). The outcomes from these investigations were highly promising, revealing that the HD-DNPEP probe displays outstanding specificity towards DNPEP.

In the following experiment, we assessed the tissue penetration capabilities of the HD-DNPEP probe after it was incubated with DNPEP (Fig. 2g–i). Upon DNPEP treatment, a clear reduction in both fluorescence and photoacoustic (PA) signals was noted as tissue thickness increased (Fig. 2g and h). Fluorescence, with its intrinsic limitations in penetrating deeper tissue layers, exhibited a marked decrease in signal strength, leading to a dramatic drop in the signal-to-background ratio (SBR). Notably, the fluorescence SBR approached baseline levels once the tissue thickness surpassed 1.0 cm, emphasizing the limited depth penetration of fluorescence-based detection techniques (Fig. 2i). Finally, we tested the photoacoustic signals of HD-DNPEP at different time points in the presence of DNPEP and found that the reaction reached its maximum value at approximately 120 min (Figure S11).

### 3.3 Response of HD-DNPEP to DNPEP in cell

The ability of the HD-DNPEP probe to detect intracellular DNPEP was evaluated through a series of experiments. First, the cytotoxicity of the HD-DNPEP probe was assessed in the RAW264.7 mouse mononuclear macrophage cell line using the thiazolyl blue tetrazolium bromide (MTT) assay. RAW264.7 cells were selected for this analysis due to their representation of macrophages involved in atherosclerosis (AS), making them a common model for immunological and inflammatory studies.

To investigate the effects of HD-DNPEP, its concentration was progressively increased from 0 to 30 μM to monitor its influence on cell survival. Notably, even at higher concentrations of HD-DNPEP, cell viability remained above 90 % (Figure S12), indicating that the compound had minimal cytotoxicity at these levels. These findings suggest that, within the tested concentration range, HD-DNPEP demonstrates low toxicity toward RAW264.7 macrophages, implying its potential safety for use in subsequent experiments.

**Fig. 3:**
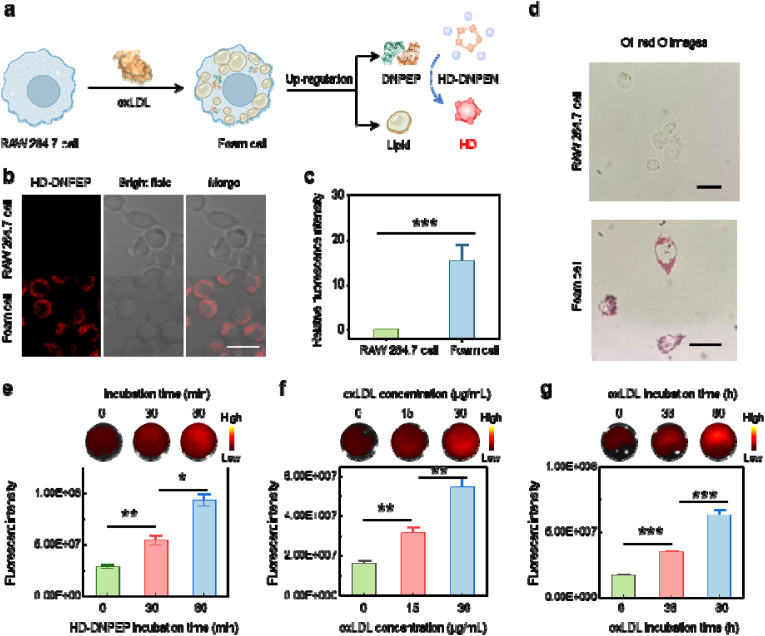
(a) Scheme of the construction of foam cells and imaging of DNPEP in cell. (b) Confocal images of HD-DNPEP (5 μM) after incubation with RAW 264.7 cell and foam cell. Scale bar = 10 μm. (c) Quantification of fluorescence intensity in (b). (d) Oil red O images of different cell. (e) Fluorescence images and quantification of HD-DNPEP after incubation with foam cell (20 μg/mL Ox-LDL for 48 h) for different times (0 to 60 min). Scale bar = 10 μm. (f) Fluorescence images and quantification of HD-DNPEP after incubation with foam cell at different progression. Incubation time of Ox-LDL: 48 h. (g) Fluorescence images and quantification of HD-DNPEP after incubation with foam cell at different progression. Ox-LDL concentration: 20 μg/mL. Results were expressed as the mean ± s.d. Statistical significance (ns: P > 0.05, ∗P < 0.05, ∗∗P < 0.01, ∗∗∗P < 0.001). (For interpretation of the references to colour in this figure legend, the reader is referred to the Web version of this article.)

RAW264.7 macrophages, a widely used murine cell line, were subjected to various treatments and categorized as follows: (i) RAW264.7 macrophages without any treatment, and (ii) foam cells, which were induced by exposing RAW264.7 macrophages to oxidized low-density lipoprotein (Ox-LDL) (Fig. 3a) [19]. After incubation with the HD-DNPEP probe, the cells were examined using confocal fluorescence microscopy (Fig. 3b). In the untreated group (RAW264.7 macrophages), no substantial fluorescence signals were observed, suggesting low intracellular DNPEP level in cell (Fig. 3c). In contrast, foam cells exhibited a noticeable increase in fluorescence intensity, confirming that the HD-DNPEP probe specifically interacted with high DNPEP level in these cells.

To further investigate the potential of the HD-DNPEP probe in monitoring foam cell development, oil red O staining was used to assess lipid accumulation. As shown in Fig. 3d, lipid accumulation within foam cells escalated as foam cell formation progressed, highlighting a relationship between foam cell maturation and lipid deposition. Following Ox-LDL induction, foam cells were treated with the HD-DNPEP probe, and a gradual increase in fluorescence intensity was observed over time. The fluorescence signal reached a steady state after 60 min of incubation (Fig. 3e), suggesting that the probe had successfully bound to DNPEP and saturated the target.

To model different stages of atherosclerosis, we varied both the concentration and duration of Ox-LDL exposure. As the concentration of Ox-LDL was elevated from 0 to 30 μg/mL, a corresponding increase in fluorescence intensity was observed (Fig. 3f). This dose-dependent relationship further confirmed the specificity of the HD-DNPEP probe for DNPEP, with higher Ox-LDL concentrations resulting in stronger fluorescence signals. Furthermore, when the duration of Ox-LDL exposure was extended from 0 to 60 h, fluorescence signals intensified over time (Fig. 3g). This suggests that as foam cells mature and accumulate more lipids, DNPEP levels increase, serving as a dynamic and reliable marker for tracking foam cell progression.

### 3.4 Response of HD-DNPEP to DNPEP in mice

**Fig. 4:**
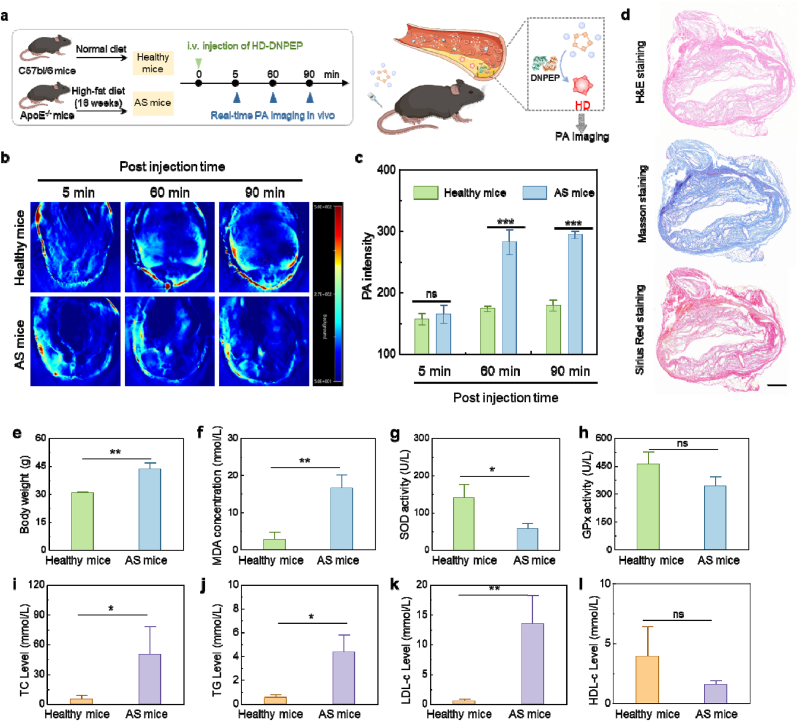
(a) Construction of healthy and AS mice, and the PA imaging process by HD-DNPEP. (b) PA images of healthy and AS mice after injection of HD-DNPEP (350 μL, 200 μM) for different times. (c) quantification of PA intensity of aortic region in (b). (d) H&E, Masson, and Sirius red staining of AS plaque. (e–h) Body weight, serum malondialdehyde (MDA), superoxide dismutase (SOD), and glutathione peroxidase (GPx) of healthy and AS mice. (i–l) Serum total cholesterol (TC), triglycerides (TG), low-density lipoprotein cholesterol (LDL-c), and high-density lipoprotein cholesterol (HDL-c) concentration of healthy and AS mice. N = 3 Results were expressed as the mean ± s.d. Statistical significance (ns: P > 0.05, ∗P < 0.05, ∗∗P < 0.01, ∗∗∗P < 0.001). (For interpretation of the references to colour in this figure legend, the reader is referred to the Web version of this article.)

In this study, C57BL/6 mice were selected as the healthy control group, while ApoE−/− mice, which were fed a high-fat diet (HFD) for 16 weeks, were used as the atherosclerotic (AS) model group (Fig. 4a) [23]. The ApoE^−/−^ mice are genetically predisposed to developing atherosclerosis, making them a suitable model for studying this cardiovascular disease.

We studied the metabolic dynamics of HD-DNPEP in mice (Figure S13). Before the injection of the probe, the fluorescence signal in the serum of mice was very weak, and the fluorescence signal in the serum was significantly enhanced 2 min after the injection of the probe. As time went by, the fluorescence signal of the probe was almost not observed 100 min after the injection of the probe, indicating that the probe could be rapidly metabolized.

To assess the imaging capabilities, both the healthy control and atherosclerotic groups were intravenously injected with HD-DNPEP, a compound designed to enhance imaging contrast. Photoacoustic (PA) imaging was employed to capture dynamic changes in tissue over time. PA images were recorded at three distinct time points following the injection: 5 min, 60 min, and 90 min (Fig. 4a).

Upon analysis of the PA signals, it was observed that, in the healthy control group, the intensity of the PA signal remained relatively stable over time. This indicates that there were no significant changes in the blood vessel signals. In contrast, the intensity of the PA signal in the atherosclerotic group significantly increased as time progressed, with a marked enhancement observed particularly at the 60- and 90-min time points. At these later time points, the PA signal intensity in the atherosclerotic group was more than two times higher than that in the control group (Fig. 4b and c). This finding suggests that the DNPEP activity in AS mice could be detectable through PA imaging.

Therefore, using the imaging agent HD-DNPEP, atherosclerotic plaques could be clearly visualized and distinguished from the healthy blood vessels. This imaging modality proved highly effective in delineating the plaques, which is crucial for the study of atherosclerosis progression and potential therapeutic interventions.

### 3.5 In vitro testing and toxicity studies

After analyzing the in vivo imaging of DNPEP, we proceeded with histological staining of blood vessels in an atherosclerotic (AS) mouse model to evaluate the extent of vascular injury. As illustrated in Fig. 4d, the aortic H&E staining of AS mice exhibited profound pathological alterations, with roughly 80 % of the vessel lumen obstructed by the buildup of atherosclerotic plaques. These findings were also corroborated by Masson and Sirius Red staining, which prominently revealed fibrous plaque deposition within the vessel walls, confirming the successful induction of the atherosclerosis model. This evidence substantiates that the AS mouse model closely mirrors human atherosclerotic conditions, serving as a dependable model for further exploration of disease mechanisms and potential therapeutic interventions.

Following this, we conducted an in-depth analysis of critical physiological and biochemical markers to distinguish the AS group from the control group. One notable observation was the body weight, a standard measure of general health, which was markedly higher in the AS mice, averaging around 43 g, compared to 30 g in the healthy controls (Fig. 4e). This weight difference likely stems from altered metabolic processes associated with atherosclerosis, which may also involve systemic inflammation and lipid accumulation.

Additionally, we assessed the redox balance in the serum of both groups, as oxidative stress is a central factor in atherosclerotic pathology. The concentration of malondialdehyde (MDA), a byproduct of lipid oxidation and a key marker of oxidative damage, was significantly elevated in the AS group, indicating an increased oxidative load compared to the healthy mice (Fig. 4f). Furthermore, the activities of vital antioxidant enzymes such as superoxide dismutase (SOD) and glutathione peroxidase (GPx) were significantly reduced in the AS mice (Fig. 4g and h), reinforcing the notion of an oxidative imbalance. These findings highlight the critical role of oxidative stress in the pathogenesis of atherosclerosis.

We also assessed the lipid of both groups by measuring the serum levels of total cholesterol (TC), triglycerides (TG), low-density lipoprotein cholesterol (LDL-c), and high-density lipoprotein cholesterol (HDL-c). The AS group exhibited significantly elevated levels of TC, TG, and LDL-c, accompanied by a reduction in HDL-c levels, as depicted in Fig. 4i–l. These lipid profile alterations are characteristic of atherosclerosis, reflecting severe disturbances in lipid homeostasis within the AS group. The elevated concentrations of atherogenic lipoproteins, combined with reduced levels of protective HDL-c, further increase the risk of plaque formation and cardiovascular complications in this model.

**Fig. 5:**
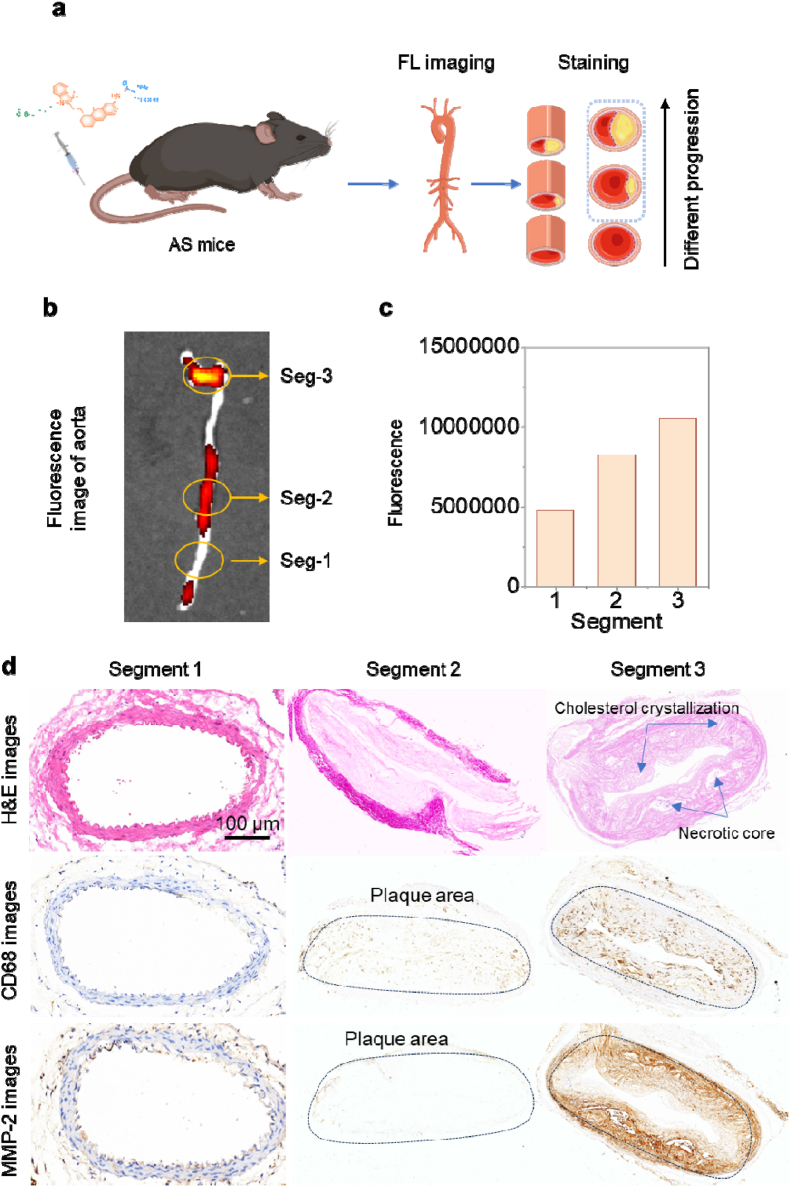
(a) Schematic representation of the experimental workflow, including intravenous administration of HD-DNPEP, aortic extraction, and fluorescence imaging. (b) Representative fluorescence images of aortic segments (Segments 1–3) showing progressive signal enhancement. (c) Quantitative analysis of fluorescence intensity across the three segments, confirming increased signal accumulation in advanced plaques. Note: Fig. 5c mainly shows the fluorescence signals corresponding to plaques at different positions of an aortic vessel. (d) Histopathological and immunohistochemical evaluation of plaque vulnerability. Top panel: H&E staining revealing atherosclerotic plaques in Segments 2 and 3, with Segment 3 exhibiting cholesterol crystals and necrotic cores. Bottom panel: Immunostaining for CD68 (macrophages) and MMP2 demonstrating elevated expression in Segments 2 and 3, correlating with plaque instability.

To elucidate the relationship between PA signals and plaque progression, a comparative tissue model was established, enabling simultaneous evaluation of vulnerable plaques, stable plaques, and normal arterial segments. This model facilitated direct comparison of plaque characteristics under controlled experimental conditions. Specifically, HD-DNPEP was intravenously administered to atherosclerotic mice, followed by aortic extraction and subsequent fluorescence imaging (Fig. 5a). Based on fluorescence intensity profiles, the aortas were systematically categorized into three distinct segments (Segments 1–3), with a progressive increase in signal intensity observed across the segments (Fig. 5b and c).

Histopathological assessment using hematoxylin and eosin (H&E) staining was performed to evaluate plaque vulnerability. The results demonstrated the presence of atherosclerotic plaques in Segments 2 and 3, whereas Segment 1 exhibited no pathological alterations (Fig. 5d). Notably, Segment 3 displayed prominent cholesterol crystal deposition and an expanded necrotic core, morphological hallmarks indicative of heightened plaque instability. Further immunohistochemical analysis involving CD68 (a macrophage marker) and MMP2 (a matrix metalloproteinase associated with extracellular matrix degradation) revealed a consistent upregulation in both markers from Segment 2 to Segment 3 (Fig. 5d). This progressive increase in macrophage infiltration and proteolytic activity strongly correlated with enhanced plaque vulnerability, supporting the notion that advanced atherosclerotic lesions exhibit greater inflammatory and destabilizing features.

The integration of fluorescence imaging with histopathological and immunohistochemical validation provided a robust framework for assessing plaque progression and vulnerability. These findings underscore the utility of multimodal imaging and molecular staining techniques in characterizing atherosclerotic disease states, offering valuable insights into the mechanisms underlying plaque destabilization.

**Fig. 6:**
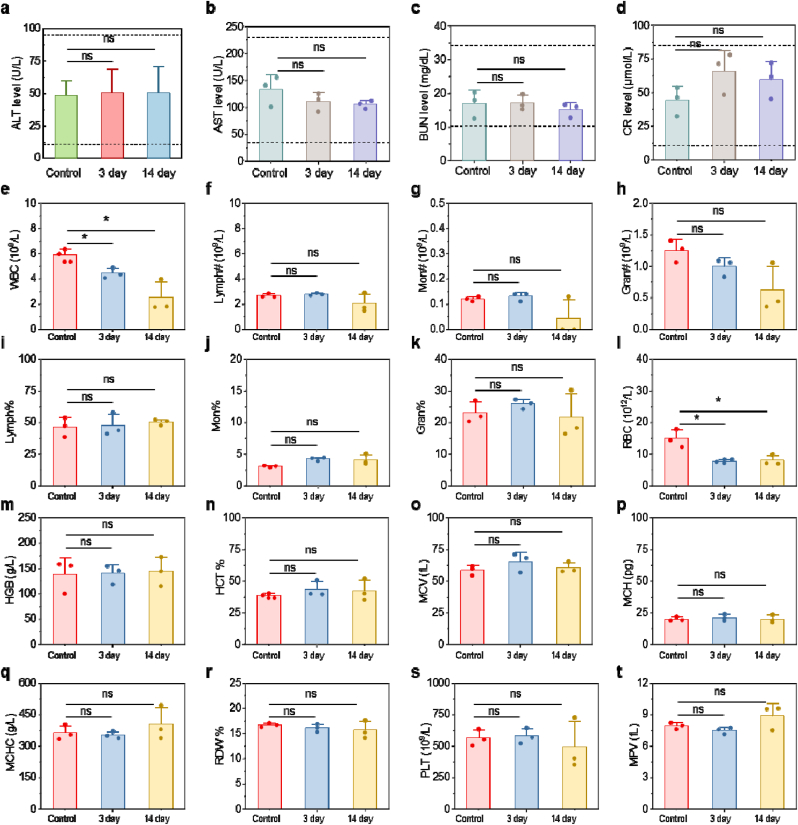
(a–d) Serum ALT, AST, BUN, and CR level of mice after injection of HD-DNPEP for different days. (e–t) Complete blood panel analysis and blood biochemistry of mice i. v. injected with HD-DNPEP for different days. Results were expressed as the mean ± s.d. Statistical significance (ns: P > 0.05, ∗P < 0.05, ∗∗P < 0.01, ∗∗∗P < 0.001).

Comprehensive hematological and biochemical analyses were performed at 3 and 14 days post-administration to evaluate potential organ toxicity. Hepatic function tests, including alanine aminotransferase (ALT) and aspartate aminotransferase (AST) levels, remained within physiological ranges, indicating preserved liver integrity (Fig. 6a and b). Similarly, renal function markers such as blood urea nitrogen (BUN) and creatinine (CR) showed no significant elevation, suggesting unimpaired kidney filtration capacity (Fig. 6c and d). Other biochemical and routine blood indicators also verified that HD-DNPEP has no obvious biological toxicity (Fig. 6e–t).

Histopathology (H&E staining) of major organs (heart, liver, spleen, lungs, kidneys) detected no damage except fatty liver vacuoles in atherosclerosis (AS) mice—likely from their high-fat diet, not HD-DNPEP (Figure S14). Overall, HD-DNPEP showed negligible toxicity, affirming their safety in this study.

### 3.6 Detection of DNPEP in mice serum

**Fig. 7:**
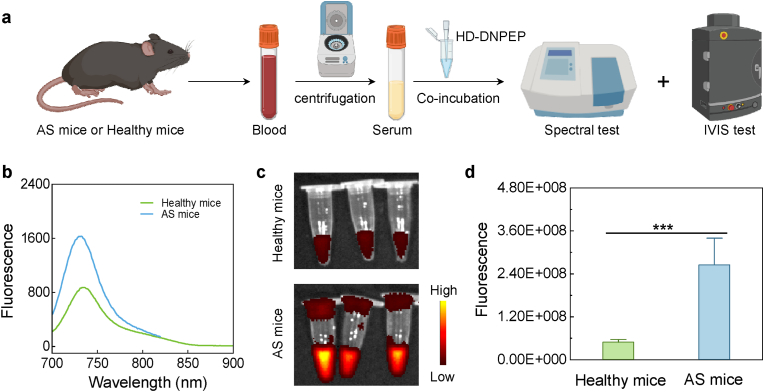
(a) Scheme of HD-DNPEP for imaging serum DNPEP activity. (b) Fluorescence spectra of HD-DNPEP after incubation with serum of healthy and AS mice. (c) fluorescence images and intensity of HD-DNPEP after incubation with serum of healthy and AS mice. Results were expressed as the mean ± s.d. Statistical significance (ns: P > 0.05, ∗P < 0.05, ∗∗P < 0.01, ∗∗∗P < 0.001).

In this study, we employed the HD-DNPEP probe as a tool to visualize and measure the levels of DNPEP in serum samples collected from both the healthy and AS groups. To carry out the experiment, we first ensured that all mice underwent an overnight fasting period to standardize the baseline serum composition. The following morning, mid-stream serum samples were collected from both the control and the AS groups. This careful protocol ensures that the results obtained are representative of the physiological conditions relevant to the research question, avoiding potential interference from recent food intake. Next, the serum samples were incubated with the HD-DNPEP probe (Fig. 7a). After incubation, the serum samples were subjected to detailed imaging analysis using fluorescence spectrum and in vivo imaging systems (IVIS), both of which are powerful tools that allow us to observe and quantify fluorescence in real-time.

Upon analyzing the images, we observed a weak fluorescence signal in the control group, which aligns with expected basal levels of DNPEP (Fig. 7b and S15). On the other hand, the AS group exhibited significantly stronger fluorescence signals, indicating an elevated presence of DNPEP in their serum (Fig. 7b and c). This suggests that DNPEP levels are notably increased in the context of atherosclerosis, providing potential evidence of its involvement in the disease process. Quantitative evaluation of the fluorescence signals further confirmed this observation, showing a statistically significant difference between the AS and control groups (Fig. 7d). These findings imply that as atherosclerotic lesions develop, there is a marked upregulation of DNPEP levels in the serum. This provides a valuable marker that could potentially be used for detection or monitoring the progression of atherosclerosis in future studies.

### 3.7 Detection of DNPEP in human samples

**Fig. 8:**
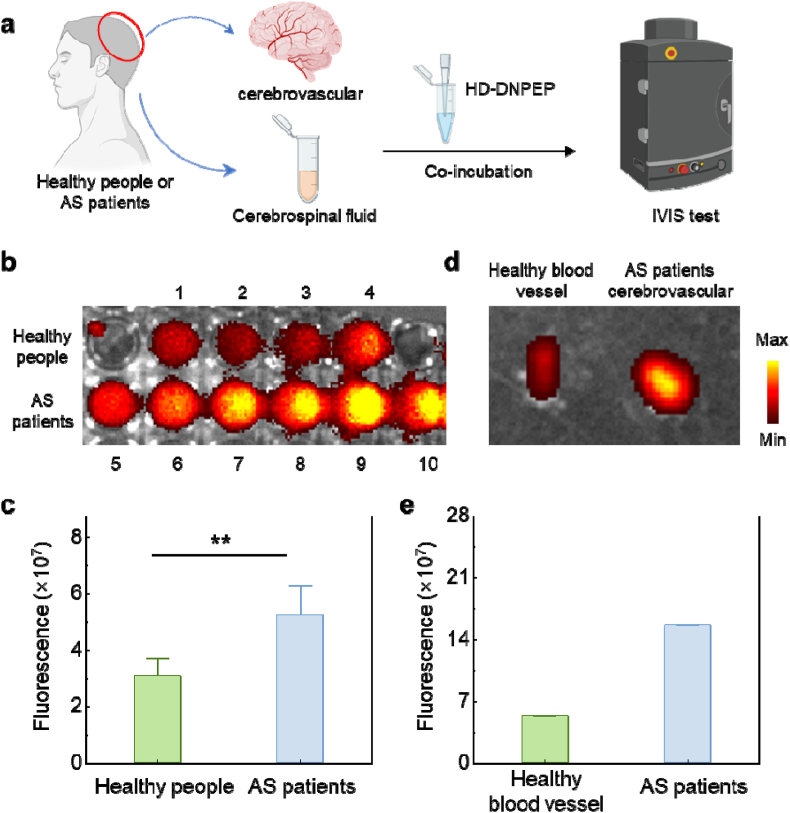
(a) Schematic diagram of HD-DNPEP used to image enzyme activity in human cerebrovascular and cerebrospinal fluid. (b) Fluorescent image of HD-DNPEP probe incubated with cerebrospinal fluid. 1–4 corresponds to different samples of healthy people, 5–10 corresponds to different samples of AS patients. (c) Fluorescent quantitative data of HD-DNPEP probe incubated with CSF. (d) Fluorescent images of HD-DNPEP probes incubated with healthy blood vessels and blood vessels of patients with AS. (e) Fluorescent quantitative data after incubation of HD-DNPEP probes with healthy blood vessels and blood vessels in patients with AS. Due to the extreme difficulty in obtaining brain tissue from both patients and healthy individuals, only one sample per group was available in this experiment.

To validate the clinical translational potential of the HD-DNPEP probe for brain vascular plaque and cerebrospinal fluid (CSF) imaging. We began by collecting CSF samples from both patients diagnosed with arteriosclerotic cerebral disease and healthy individuals. The HD-DNPEP probe was then incubated with the CSF samples, followed by imaging and quantitative analysis using the IVIS imaging system (Fig. 8a). The results revealed that the fluorescence signal from the HD-DNPEP probe was significantly stronger in the CSF from AS patients compared to healthy controls (Fig. 8b and c). This enhanced fluorescence signal indicates elevated DNPEP enzyme activity in the CSF of AS patients, supporting the notion that the HD-DNPEP probe can effectively detect enzyme activity linked to atherosclerosis diseases.

Next, we collected cerebral vessels from both AS patients (experimental group) and patients with benign brain tumors located in the ventricles (control group). These vessels were incubated with the HD-DNPEP probe, and fluorescence imaging was again performed (Fig. 8d and e). The findings demonstrated that the cerebral vessels from AS patients exhibited a stronger fluorescence signal compared to the control group, further supporting the increased activity of DNPEP in the cerebral vasculature of AS patients. These results provide compelling evidence that the HD-DNPEP probe is capable of detecting elevated DNPEP activity in both CSF and cerebral vasculature.

## 4 Conclusion

The HD-DNPEP probe, synthesized by condensing HD-SO_3_H with DNPEP substrates, demonstrated high sensitivity and specificity for DNPEP detection, showing "turn-on" fluorescent and PA signals. In cellular studies, the probe was non-toxic (≤30 μM) and effectively detected DNPEP in foam cells, correlating fluorescence intensity with atherosclerosis progression. In vivo, it distinguished atherosclerotic plaques via PA imaging without toxicity and identified elevated serum DNPEP levels in mice, suggesting diagnostic potential. Additionally, higher DNPEP levels in the cerebral vasculature and CSF of atherosclerosis patients indicate its utility as a biomarker for cerebrovascular arteriosclerosis. The probe could enable non-invasive disease monitoring and treatment evaluation, highlighting its promise for clinical diagnostics and research.

## CRediT authorship contribution statement

**Chenhui Zhou:** Formal analysis, Data curation, Conceptualization. **Fangkun Yang:** Software, Resources, Project administration, Methodology. **Chunyan Li:** Supervision, Software, Resources. **Hengyi Mao:** Supervision, Software, Resources. **Kai Wang:** Investigation, Funding acquisition. **Jinhui Shang:** Visualization, Validation, Supervision. **Xiang Gao:** Writing – review & editing, Writing – original draft, Supervision. **Wenming He:** Writing – review & editing, Writing – original draft, Visualization, Validation.

## Funding

This work was supported by grants from the Key Laboratory of Precision Prevention and Treatment for Atherosclerotic Diseases of Zhejiang Province, Cardiovascular Disease Clinical Medical Research Center of Ningbo, 10.13039/501100001809National Natural Science Foundation of China (82200489), the Major Project of Science and Technology Innovation 2025 in Ningbo, China (Grant No. 2021Z134), the medicine and health science and technology projects of Zhejiang province (2023KY265), Ningbo Top Medical and Health Research Program (No. 2022020304), the Key research and development project of Zhejiang Province, China (No. 2021C03096 and 2023C04017) and the Innovation Yongjiang 2035′ Key R&D Programme (2024H010). The authors extend their gratitude to Ms. Yuan Zhou from Scientific Compass (www.shiyanjia.com) for providing invaluable assistance with the confocal analysis.

## Declaration of competing interest

The authors declare that they have no known competing financial interests or personal relationships that could have appeared to influence the work reported in this paper.

## Data Availability

Data will be made available on request.

## Appendix A Supplementary data

General information about materials, experimental details, including detailed synthetic procedures, ^1^H NMR, ^13^C NMR, and other characterizations of the HD-DNPEP, and supplemental in vitro characterization.
