# Supplementary material for: Detection of aspartyl aminopeptidase in atherosclerosis mice and clinical sample using an optical probe
Source: Mater Today Bio. 2025 Oct 8;35:102391. doi: 10.1016/j.mtbio.2025.102391 (PMC12546970; doi:10.1016/j.mtbio.2025.102391)
Supplement: Multimedia component 1 [file mmc1.doc]

Supplementary Information (SI)

**Detection of Aspartyl Aminopeptidase In Atherosclerosis Mice and Clinical Sample Using an Optical Probe**

Chenhui Zhou^1,†^, Fangkun Yang^3,4,†^, Chunyan Li^3,†^, Hengyi Mao^3^, Kai Wang^2,*^, Jinhui Shang^4,*^, Xiang Gao^1,*^, Wenming He^3,4,*^

^1^Department of Neurosurgery, The First Affiliated Hospital of Ningbo University, School of Medicine, Ningbo University, Ningbo, China.

^2^State Key Laboratory of Pharmaceutical Biotechnology, School of Life Sciences, Nanjing University, Nanjing, 210023, China.

^3^Department of Cardiology, The First Affiliated Hospital of Ningbo University, School of Medicine, Ningbo University, Ningbo, China.

^4^Key Laboratory of Precision Prevention and Treatment for Atherosclerotic Diseases of Zhejiang Province, Ningbo, China.

^†^These authors have contributed equally to this work.

*Corresponding author E-mail: hyxj0309@163.com (K. Wang); jhshang@126.com (J.H. Shang); qinyuecui@163.com (X. Gao); fyhewenming@nbu.edu.cn (W.M. He).

Supplementary Experiment Section

All nuclear magnetic resonance analyses were conducted on a Bruker DRX-400 spectrometer operating at 400 MHz for proton observation. Following lyophilization processing with a (SCIENTZ-18N, Ningbo Scientz Biotechnology Co., Ltd., China) at-80 °C. The pH quantification was performed using a PHS-25 pH meter. All aqueous solutions were prepared with ultrapure water produced by Milli-Q IQ 7000 (Billerica, MA, USA). UV-vis absorption spectra were acquired using a Shimadzu UV-2600 spectrophotometer (Kyoto, Japan) and fluorescence emission profiles were recorded on a Hitachi F-7100 spectrophotometer (Kyoto, Japan), respectively. The mass spectra were acquired from Agilent 7250 GC/Q-TOF 7000D (Santa Clara, CA, USA). The cell cytotoxicity was measured using a Tecan microplate reader (Männedorf, Switzerland). In vivo imaging was executed on an IVIS Lumina XR small animal optical imaging system (Perkin-Elmer, Waltham, MA, USA). The chemical synthesis, in vitro activity assay, and imaging in living cells and mouse models are systematically documented in the Supplementary Information.

**Synthesis of HD-DNPEP.** 3-Nitrophenol (347 mg, 2.5 mmol) and K_2_CO_3_ (345 mg, 2.5 mmol) were dissolved in 15 mL CH_3_CN in a flask, and the mixture was stirred at room temperature under an N_2_ atmosphere. Then, IR780**-**SO_3_H (782 mg, 1 mmol) in CH_3_CN (5 mL) was introduced to the mixture via a syringe and the reaction mixture was stirred at room temperature for 4 h. The solvent was then evaporated under reduced pressure and the precipitate was dissolved in CH_2_Cl_2_, followed by washing with water three times. The residue obtained by evaporation was dispersed in 20 mL CH_3_OH for further use in the next step. SnCl_2_ (4 g, 20 mmol) was dissolved in concentrated HCl (4 mL), followed by adding the above solution under an N_2_ atmosphere. The reaction solution was heated to 70 ° C and stirred for 6 h. Then, the solution was extracted by saturated NaCl and CH_2_Cl_2_. The solvent was removed by evaporation under reduced pressure, and the residue was purified by silica gel chromatography with CH_2_Cl_2_/CH_3_OH (100/1 to 20/1, v/v) as eluent, affording HD-SO_3_H as a dark green solid (yield = 23%).

Boc-peptide (50 mg), HATU (70 mg), and DIPEA (40 μL) were dissolved in CH_3_CN (1 mL) and CH_2_Cl_2_ (4 mL) with stirring for 60 min. Then, HD-SO_3_H (20 mg) in CH_2_Cl_2_ was introduced, and the reaction mixture was further stirred at room temperature for 12 h. Then, the solution was extracted with saturated brine and CH_2_Cl_2_, and the organic solvent was evaporated under reduced pressure at 40°C. Finally, the residue was purified by silica gel chromatography with CH_2_Cl_2_/CH_3_OH (v/v = 50/3) as eluent to obtain Boc-HD-DNPEP as a blue solid (yield = 42%).

Then, Boc-HD-DNPEP (10 mg) was dissolved in CH_2_Cl_2_ (2 mL) and CF_3_COOH (2 mL) with stirring at room temperature. Then, 50 μL triisopropylsilane was added to the solution, and the solution was stirred for 30 min. After removing the solvent under reduced pressure, the residue was purified by silica gel chromatography with CH_2_Cl_2_/CH_3_OH (v/v = 10/1) as eluent to obtain the HD-DNPEP as a blue solid (yield = 98%). ^1^H NMR (400 MHz, MeOD) δ 10.64, 8.88, 8.51, 8.47, 7.81, 7.78, 7.78, 7.77, 7.75, 7.73, 7.64, 7.62, 7.57, 7.55, 7.54, 7.52, 7.51, 7.49, 7.47, 7.45, 7.43, 6.59, 6.58, 6.56, 4.45, 4.43, 4.41, 3.97, 3.95, 2.72, 2.71, 2.69, 2.68, 2.66, 2.64, 2.63, 2.61, 2.60, 2.58, 2.16, 2.14, 2.12, 2.10, 1.95, 1.93, 1.91, 1.89, 1.88, 1.86, 1.84, 1.82, 1.80, 1.71.

**Cellular experiments**

RAW 264.7 macrophages were cultured in DMEM medium supplemented with 10% fetal bovine serum (FBS) and 1% antibiotics (penicillin-streptomycin).

For cell fluorescent imaging, In Vivo Imaging system (IVIS) was used: Imaging parameters: Fluorescence; Excitation wavelength: 640 nm Filter: Cy5.5 channel (690–770 nm); Field of view: B; Software: Living Image 4.0 software.

**Animal experiments**

ApoE^-/-^ (apolipoprotein E knockout) mice (5–8 weeks, male) were purchased from GemPharmatech Co., Ltd.

For PA imaging of aorta ex vivo. The aorta of atherosclerotic mice was separated after i.v. injection of HD-DNPEP for 3 h. The ex vivo fluorescent image of the aorta was recorded by an IVIS spectral imaging system (Lumina XR), and PA images were obtained.

For photoacoustic imaging, a commercial small animal photoacoustic imaging system was used: Model: inVision 256-TF (iThera Medical GmbH, Munich, Germany); Optical parametric oscillator (OPO): Nd: YAG laser; Excitation pulses: 9 ns; Wavelengths range: 680 nm to 980 nm; Repetition rate: 10 Hz; Wavelength tuning speed:10 ms; Peak pulse energy: 100 mJ; Center frequency of ultrasound transducers: 5 MHz (60% bandwidth).

The aorta was separated from atherosclerotic mice and embedded in optimal cutting temperature (OCT) compound directly, frozen at -80°C for 30 minutes, then sliced at a thickness of 10 μm.

For aorta hematoxylin-eosin (H&E) staining, frozen sections of the aorta were stained with 10 μL hematoxylin for 5 min at room temperature, then those sections were rinsed three times with deionized water and stained with 10 μL eosin for 10 s. Next, those sections were dehydrated three times with 10% ethanol and added one drop of neutral balsam. Finally, those sections were covered with a coverslip and scanned by the digital slice scanning system.

For PA imaging of healthy mice and atherosclerotic mice, C57bl/6 mice were deemed as healthy mice, and ApoE^-/-^ mice were fed with a high-fat diet (HFD) to construct atherosclerotic mice. Those mice of different groups were i.v. injected with PBS containing HD-DNPEP (350 μL, 200 μM), and PA images of each group were recorded at 695 nm at different time points after injection.

**Extraction of Cerebral Vessels from Patients**

The cerebral vessels from the experimental group are obtained during surgery from patients diagnosed with intracerebral hemorrhage caused by arteriosclerotic cerebrovascular disease. These vessels are preserved during the surgical procedure for later use in the experiment.

The cerebral vessels from the control group are obtained from patients undergoing cortical shunting for benign brain tumors located in the ventricles. These patients require the procedure to relieve pressure, and the vessels are collected during the surgery.

**Extraction of Cerebrospinal Fluid (CSF) from patients**

The cerebrospinal fluid (CSF) is collected from patients who undergo routine lumbar puncture procedures in clinical practice. The CSF samples are collected during this procedure for use in the experiment.

**Statistics and data analysis.** Results were expressed as the mean ± s.d. Statistical significance (*P < 0.05, **P < 0.01, ***P < 0.001) was performed by one-way analysis of variance (ANOVA).

Figure S1. Synthetic route of HD-DNPEP.


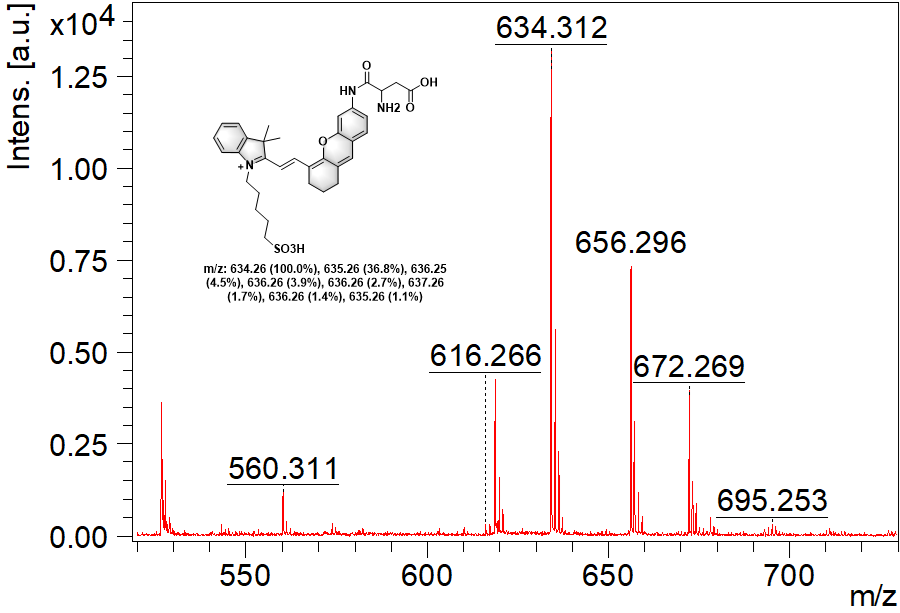


Figure S2. MS spectrum of HD-DNPEP

Figure S3. ^1^H-NMR spectrum of HD-DNPEP

Figure S4. ^13^C-NMR spectrum of HD-DNPEP


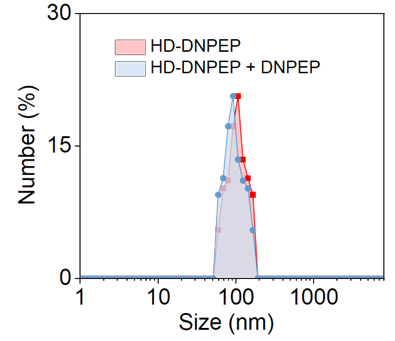


Figure S5. DLS analysis of HD-DNPEP before and after incubation with DNPEP.


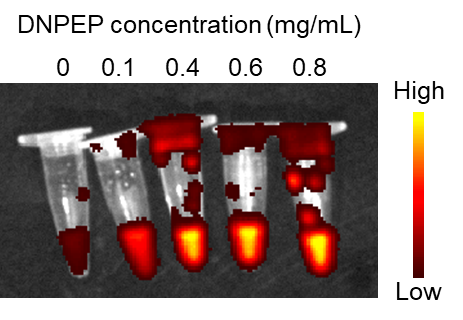


Figure S6. Fluorescence images of HD-DNPEP after incubation with different concentrations of DNPEP.


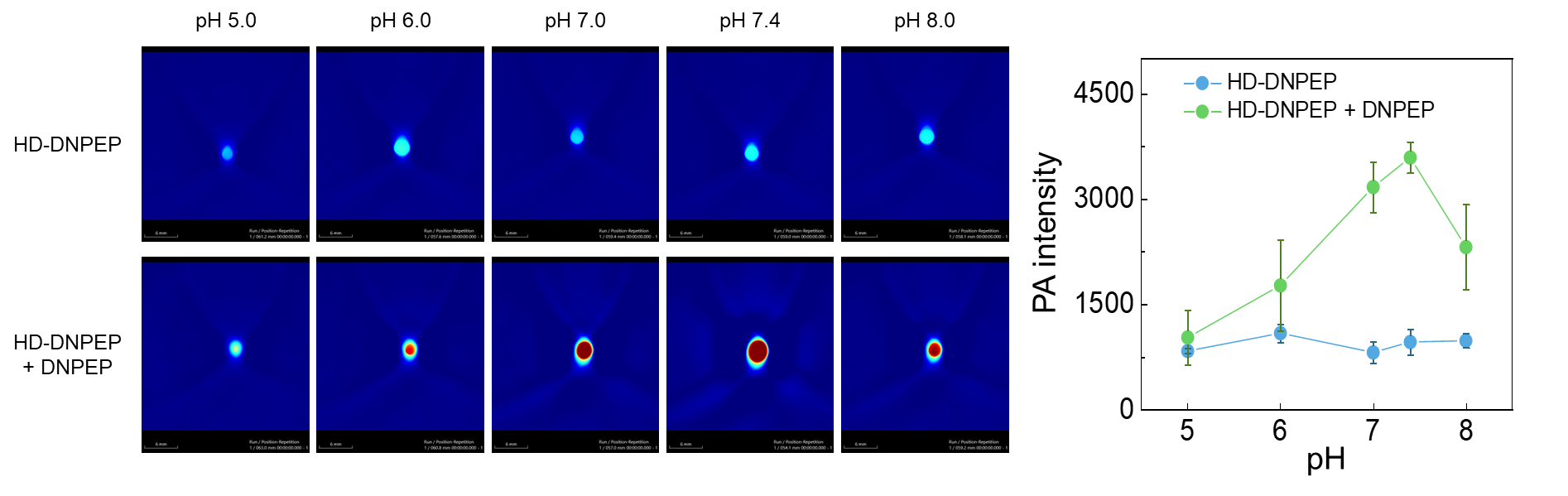


Figure S7. PA images and intensity of HD-DNPEP before and after incubation with DNPEP at different pH buffers.


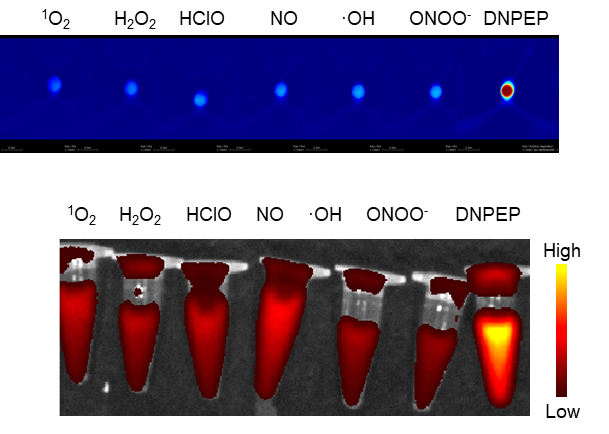


Figure S8. PA and fluorescence images of HD-DNPEP after incubation with various species. DNPEP: 0.5 mg/mL, other species: 50 μM.


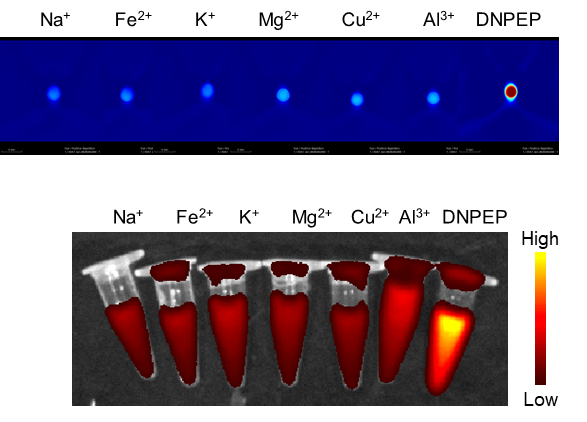


Figure S9. PA and fluorescence images of HD-DNPEP after incubation with DNPEP and various metal ions. DNPEP: 0.5 mg/mL, other metal ions: 50 μM.


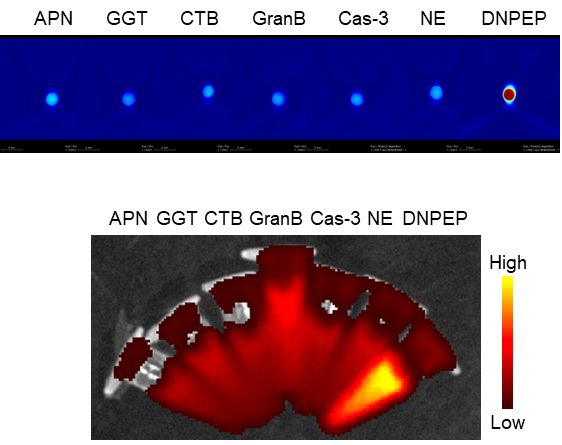


Figure S10. PA and fluorescence images of HD-DNPEP after incubation different enzymes.


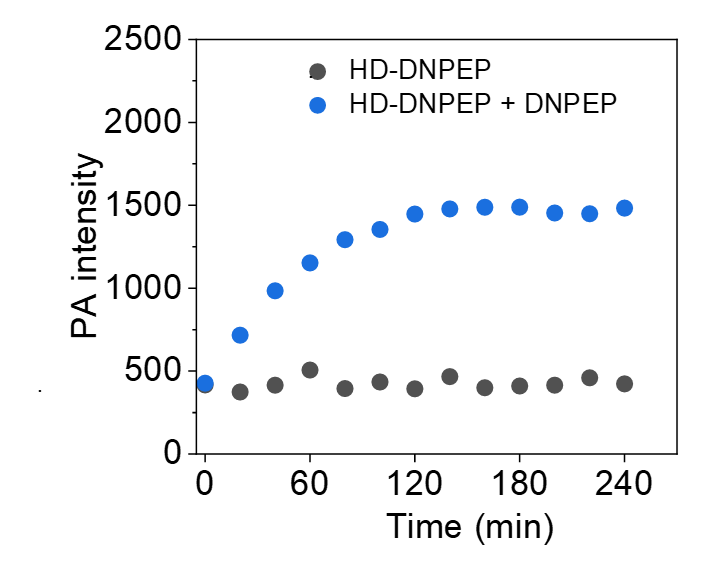


Figure S11. Kinetic study of HD-DNPEP to DNPEP.


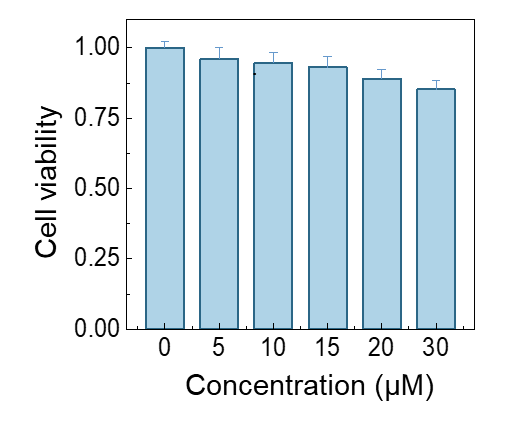


Figure S12. Cell viability of RAW 264.7 cell after incubation with different concentrations of HD-DNPEP.


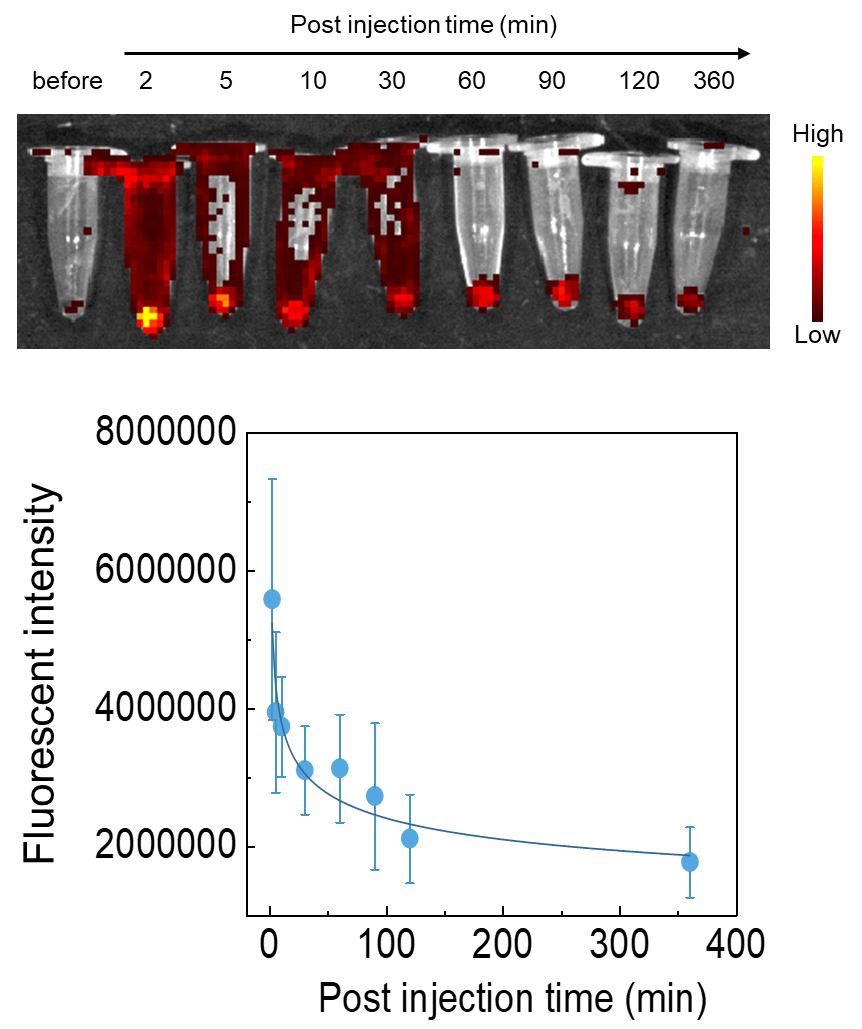


Figure S13. Blood fluorescence images and intensity before and after i.v. injection of HD-DNPEP into living mice.


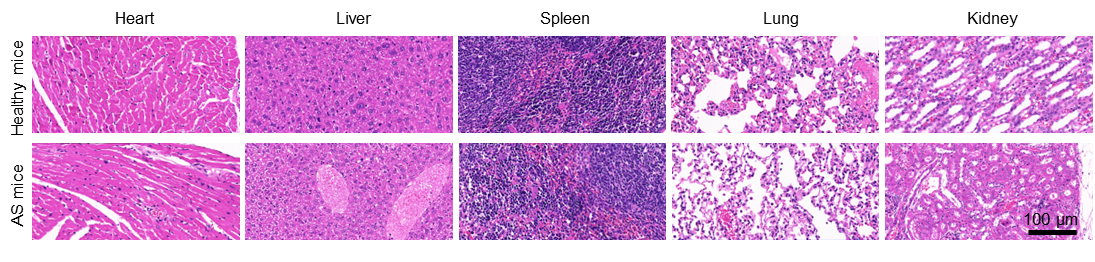


Figure S14. H&E-staining images of major organs from control mice, and mice i. v. injected with HD-DNPEP


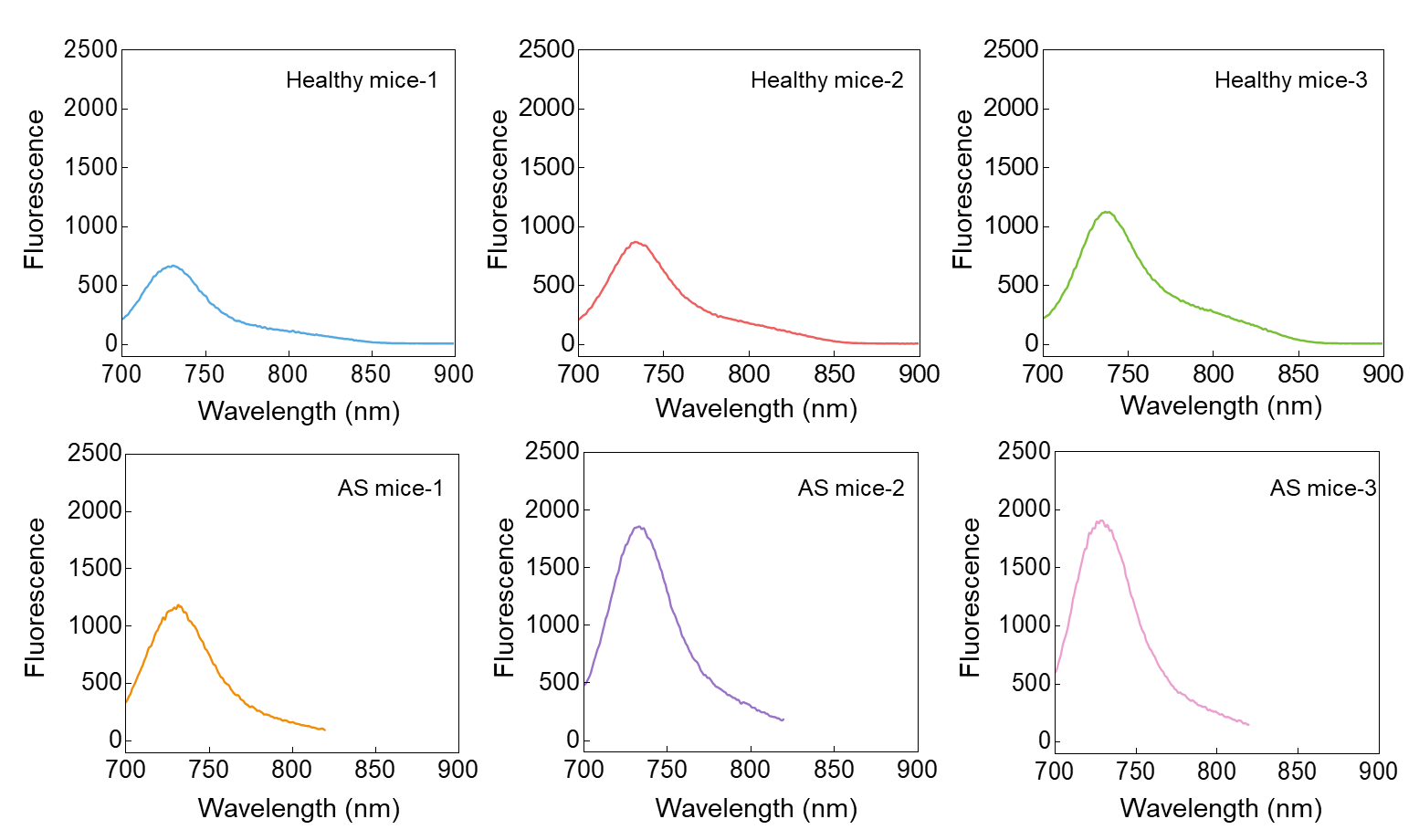


Figure S15. Fluorescence spectra of serum after incubation with HD-DNPEP
